# Histological Improvement and Cytokine Levels Reduction in Patients with Oral Lichen Planus after Photobiomodulation Therapy

**DOI:** 10.3390/biomedicines12102300

**Published:** 2024-10-10

**Authors:** Maria Zaharieva Mutafchieva, Milena Nenkova Draganova, Georgi Tomchev Tomov

**Affiliations:** 1 Department of Periodontology and Oral Mucosa Diseases, Faculty of Dental Medicine, Medical University of Plovdiv, 4000 Plovdiv, Bulgaria; 2 Department of Medical Biology, Faculty of Medicine, Medical University of Plovdiv, 4000 Plovdiv, Bulgaria; 3 Research Institute, Medical University of Plovdiv, 4000 Plovdiv, Bulgaria; 4 Department of Healthcare and Social Work, New Bulgarian University, 1618 Sofia, Bulgaria; dr.g.tomov@gmail.com

**Keywords:** OLP, PBM therapy, cytokines—IL-1β, IL-6 and TNF-α, histological examination

## Abstract

**Background:** Oral lichen planus (OLP) is a mucocutaneous disease associated with the formation of symptomatic lesions in the mouth that are often refractory to treatment. An as-yet-unknown antigen triggers an inflammatory reaction in which various immune and non-immune cells release multiple cytokines that contribute to disease progression. The ability of photobiomodulation (PBM) to reduce the symptoms and signs of the disease has been shown, but little is known about its molecular and cellular effects. The aim of this study was to evaluate changes in pro-inflammatory cytokine levels and in histological findings in OLP patients treated with photobiomodulation therapy. **Methods:** Twenty OLP patients underwent PBM with diode laser (810 nm), (0.50 W, 30 s, 1.2 J/cm^2^), 3 times weekly for a month. Pain level and clinical scores of lesions were recorded before and after therapy. Salivary levels of IL-1β, IL-6, and TNF-α in OLP patients were measured before and after PBM and compared with those of 10 healthy controls. Biopsies were taken at the beginning and end of treatment to assess pathomorphological changes. **Results:** PBM significantly reduced the level of pain and clinical scores of the lesions. Salivary levels of IL-1β, IL-6, and TNF-α in OLP patients were significantly higher compared to those in healthy controls and decreased after therapy. 60% of the post-treatment OLP biopsies demonstrated histological improvement, characterized by inflammatory infiltrate reduction (50%), epithelial hyperplasia reduction (30%), epithelial thickening (15%), or epidermal-dermal attachment repair (5%). **Conclusion:** The effectiveness of PBM therapy in OLP patients was confirmed at the clinical, molecular, and histomorphological levels.

## 1. Introduction

Lichen planus is a chronic mucocutaneous disease that can affect the skin, various mucous membranes, scalp, and nails. Oral lesions (Oral lichen planus—OLP) are found in about 2/3 of patients, and in up to 25% of cases they are the only manifestation of the disease [1]. Clinically, OLP presents in different clinical forms—keratotic (reticular, papular, plaque-like) and non-keratotic (atrophic, bullous, and erosive forms) [1,2]. Wickham striae, representing white keratotic lines in a lace-like pattern, are considered a hallmark of the disease [2]. In symptomatic OLP, patients complain of a burning sensation to severe pain [3]. Furthermore, OLP is included in the group of oral potentially malignant disorders (OPMDs) [4].

To date, the etiopathogenesis of OLP is not fully understood. The overall consensus is that it is an immune-mediated disease, initiated by the expression of a given antigen on the surface of the basal keratinocytes of the oral epithelium. A number of viruses, microbial antigens, fungi, self-peptides, or heat shock proteins have been extensively investigated as putative triggers, and yet the specific etiologic factor remains ambiguous [5,6]. Several phases in the pathogenesis are distinguished—trigger event, release of immunoregulatory cytokines and chemokines, activation of vascular adhesion molecules, migration, recruitment, and activation of T lymphocytes at the diseased site with consequent cytotoxic response, inducing apoptosis of the basal keratinocytes [7]. The main lymphocytes involved are thought to be cytotoxic CD8+ T-Ly, which have been found within the epithelium and adjacent to apoptotic keratinocytes, while CD4+ T-Ly are the predominant cell type in lamina propria [8]. These and other cell types, such as degranulated mast cells [9], macrophages, Langerhans cells, and basal keratinocytes release a multitude of pro-inflammatory and chemotactic cytokines (TNF-α, IL-1β, IL-6, IL-12, and G-CSF), chymase, tryptase, and matrix metalloproteinases (MMP) [5]. This abundance of cytokines orchestrates a complex interplay between cells and leads to the development of a chronic, dysregulated, harmful immune response, culminating in KC cell death.

Oral lichen planus often presents a significant diagnostic challenge, since there are several diseases that mimic OLP both clinically and pathomorphologically [2,10]. Histological criteria for OLP were first proposed by the World Health Organization (WHO) in 1978 [11] and then modified by Van der Meij in 2003 [12]. In 2016, the American Academy of Oral and Maxillofacial Pathology updated these criteria to include band-like, predominately lymphocytic infiltrate, confined to the epithelium–lamina propria interface; basal cell liquefactive (hydropic) degeneration; lymphocytic exocytosis; absence of epithelial dysplasia; absence of verrucous epithelial architectural change [10].

Treatment is another provocation for clinicians dealing with patients with OLP, as there is no universally effective or curative therapy. Among the pharmacological agents used in OLP treatment are corticosteroids, retinoids, calcineurin inhibitors, dapsone, hydroxychloroquine, and other immunosuppressants. Topical corticosteroids (CS) are the first choice; however, they are also associated with side effects such as thinning of the oral mucosa, oral candidiasis, etc. [13]. Phototherapy applied in OLP includes UVA- and UVB light (not used nowadays), ablative and non-ablative (photobiomodulation and anti-microbial photodynamic therapy) laser approaches.

Photobiomodulation (PBM), formerly known as low-level laser therapy (LLLT), is the application of a laser or LED to favorably affect cellular metabolism [7]. It is a non-thermal approach, which should not cause any observable tissue changes [14]. Growing scientific evidence has confirmed that it affects mitochondria inside the cell to stimulate adenosine triphosphate (ATP) production, controls ROS (reactive oxygen species), and lowers oxidative stress [14]. It has been shown that PBM reduces inflammation by lowering prostaglandin E2, prostaglandin-endoperoxide synthase 2, interleukin 1 beta (IL-1β), and tumor necrosis factor-alpha (TNF-α) [14]. In addition, PBM reduces pain intensity by stimulating the B endorphins, reducing the release of pain mediators (histamine, bradykinins, substance P, acetylcholine), decreasing the activities of C fiber by blocking depolarization, stimulating replenishment of neurotransmitters at synaptic level [7,13]. Finally, laser irradiation promotes wound healing by increasing the secretion of growth hormone, activating the TGF-β family of cytokines (transforming growth factors-β), promoting the proliferation of fibroblasts, keratinocytes, osteoblasts, and chondrocytes, matrix synthesis, angiogenesis, and vascular remodeling [15,16].

Over the past three decades, a great number of articles have been published addressing the utility of PBM therapy in the management of OLP. Del Vecchio et al. conducted a database search to retrieve all relevant studies released between 1992 and 2019 [7]. All 44 studies meeting the authors’ inclusion criteria reported positive effects of PBM [7]. Later, in 2022, another systematic review article evaluated the effectiveness of PBM in atrophic-erosive forms of OLP by extracting and summarizing data from 7 related studies and concluded that this type of therapy successfully improves the signs and symptoms of these lesions with no known side effects [13]. Different laser types (diode laser, Nd–YAG, He–Ne laser, etc.) with wavelength windows between 630 nm and 1064 nm and wide heterogeneity in the laser parameters have been used among the analyzed studies.

Interestingly, multiple studies have compared the effectiveness of corticosteroids (CS) therapy and PBM in improving the signs and symptoms of OLP and demonstrated similar results in the groups, favoring the latter due to fewer or no side effects known [14,17,18,19,20]. Conversely, other authors found that CS were significantly better at treating these patients [21,22]. Nevertheless, the general consensus is that PBM can be used successfully in cases where CS are ineffective or contraindicated due to comorbidity.

However, the PBM efficacy statement is primarily based on clinical outcomes—improvement in subjective symptoms and clinical presentation [13]. Few reports have provided molecular or pathomorphological evidence for the efficacy of the laser biomodulation in OLP. In this regard, Mohamed RK et al. investigated the change in salivary malondialdehyde (MDA) levels in OLP patients after PBM treatment [14]. MDA has been regarded as a valid biomarker for the levels of oxidative stress, and increased serum and salivary concentrations of this bioproduct have been detected in OLP patients [23]. The authors found that laser light therapy is equivalent to corticosteroids in reducing MDA levels and thus—oxidative stress [14]. This may be of particular benefit since not only the development but also malignant transformation of OLP have been linked to a condition of oxidative stress [14].

As mentioned above, the milieu of cytokines secreted by immune and non-immune cells at the lesional site in OLP contributes to the chronicity of the disease. However, few studies in the literature evaluated the modulation of cytokines involved in OLP pathogenesis after treatment. Moreover, most of the articles related to the topic addressed the effects of topical or systemic corticosteroids on cytokine levels [24,25]. Abboud CS et al. (2021) found no modulation in serum and salivary levels of IL-1β, -4, -6, -10, 17A, and TNF-α in OLP patients after PBM, although significant improvement in pain and clinical performance was achieved [3]. In addition, Othman NA et al. also showed no significant reduction in TNF-α levels after 970 nm laser irradiation in OLP patients [21]. The reported poor results are surprising since PBM has been shown to decrease cytokine levels in experimental models of inflammatory diseases [26].

Salivary levels of IL-1β [27], TNF-α [28,29], and IL-6 [28,30] have previously been reported to be elevated in OLP patients compared with healthy controls, suggesting that these cytokines may be used as disease-related biomarkers for monitoring of disease activity and response to various therapies. In our previous in vitro experiment [31], we found that an 810 nm diode laser (0.5 W, 30 s, 1.2 J/cm^2^) significantly reduced the levels of IL-1β and IL-6 secreted by lipopolysaccharide (LPS)-stimulated peripheral blood mononuclear cells (PBMC). Тherefore, in the present study, we applied the same therapeutic protocol to evaluate the effects of PBM therapy on IL-1β, IL-6, and TNF-α levels in OLP patients.

More importantly, addressing the topic of the effectiveness of PBM in the treatment of OLP patients, it would be of particular interest to reveal the resulting histological changes. Тo the best of our knowledge, there are no studies in this field in the literature.

Therefore, the aim of this study was to evaluate changes in pro-inflammatory cytokine levels and in histological findings in OLP patients after PBM therapy.

## 2. Materials and Methods

### 2.1. Study Design

This was a prospective clinical observational study. Twenty patients with Oral lichen planus were selected among those attending the Department of Periodontology and Oral Mucosal Diseases of the Faculty of Dental Medicine, Medical University of Plovdiv, Bulgaria. The diagnosis of OLP was confirmed by histological examination. A full explanation of the treatment and study objectives, including a patient information leaflet, was then provided, and informed written consent was obtained from all recruited patients. Additionally, another informed written consent was obtained from all the subjects regarding publishing their clinical photos and the study in a scientific peer-reviewed journal. At baseline (14 days after the first biopsy), the number of the lesions as well as their type, size, location, and score (0–5) according to the Thongprasom Scoring Sign System (TSSS) were recorded for all participants. Self-reported pain level was assessed using a visual analogue scale (VAS). Unstimulated whole saliva was collected from all twenty patients to evaluate the levels of the pro-inflammatory cytokines IL-1β, IL-6, and TNF-α by the ELISA method. The diagnostic glass slides were used to record initial (pre-treatment) histological findings for all participants. All OLP patients were then treated with PBM therapy with the protocol described below for one month. At the end of treatment, clinical outcomes were assessed by re-determining the pain level values and clinical scores of the lesions and comparing them with those before therapy. Control saliva and biopsy samples were taken two days after the last 12th treatment session from all OLP patients to reveal changes in cytokine levels and histological findings in response to PBM therapy. Meanwhile, unstimulated whole saliva was also collected from 10 volunteers to determine differences in cytokine levels between OLP patients and healthy subjects.

The null hypothesis of the study was that there is no statistically significant difference in the pain level scores, clinical signs of the lesions, or salivary concentration of pro-inflammatory cytokines of OLP patients before and after PBM therapy.

All clinical procedures were conducted at the Department of Periodontology and Oral Mucosa Diseases, Faculty of Dental Medicine, Medical University of Plovdiv, while laboratory and histological examinations were performed at the Department of Medical Biology and the Department of General and Clinical Pathology respectively, Faculty of Medicine, Medical University of Plovdiv.

The study was conducted in accordance with the Declaration of Helsinki. The research protocol was approved by the Ethics Committee of Medical University Plovdiv (R3716/07.10.2014).

#### 2.1.1. Research Focused Questions

- Can 810 nm PBM therapy improve the symptoms and clinical signs of OLP?
- Are the levels of the pro-inflammatory cytokines IL-1β, IL-6, and TNF-α significantly different in OLP patients than in healthy controls, and if so, can 810 nm PBM irradiation correct this abnormality?
- What histological changes does PBM therapy induce in OLP patients?

#### 2.1.2. Research Contingent

- Intervention group—20 patients with oral lichen planus
- Saliva from 10 voluntary donors without oral or systemic diseases was used as a control for cytokine levels in health

#### 2.1.3. Inclusion Criteria

- Patients with symptomatic lesions of any type of clinical forms of OLP—reticular, papular, plaque-like, atrophic, bullous, or erosive form
- OLP patients of both genders (female and male) aged ≥ 18-year-old
- Patients with histologically confirmed diagnosis OLP
- Patients who agreed with study design
- 10 age- (≥18-year-old; mean age 54.9) and sex (8 women: 2 men)-matched healthy volunteers who came for a routine dental examination and agreed to donate saliva samples. They all signed an informed consent. Inclusion in the study required a clinical finding of no oral lesions, periodontal disease or endodontic problems and a history of no systemic diseases

All subjects from the test (patients) group and from the control group were from the population of Bulgaria

#### 2.1.4. Exclusion Criteria

- Subjects with oral lichenoid reactions (OLR) suspicion—unilateral lesions with direct topographic relationship to amalgam fillings/dental restoration(s); history of a temporal association between the introduction of some drug and the onset of the disease; history of past transplantation; histologically: detection of polymorphic inflammatory infiltrate containing eosinophils, plasma cells, and neutrophils; inflammatory infiltrate in deeper areas; focal perivascular infiltrate; tertiary lymphoid follicles [32].
- History of corticosteroids or non-steroidal anti-inflammatory drug treatment in the last 1 month.
- Signs of epithelial dysplasia in the histological sections.

### 2.2. Collection of Samples

#### 2.2.1. Saliva Samples Collection

Whole unstimulated saliva for cytokine measurement was collected from all 20 OLP patients and 10 healthy volunteers between 08:00 and 10:00 AM. Subjects were instructed to rinse the mouth with water and two minutes later to spit the saliva without stimulation into sterile 10 mL containers. A total of 5 mL of saliva was collected over 10 min. Samples were stored on ice for no more than 1 h after collection. Saliva was then centrifuged for 10 min at 1500 rpm at 4 °C to remove debris and cellular material. The supernatant was aliquoted into sterile tubes and stored at −80 °C until analysis.

#### 2.2.2. Incisional Biopsy with Er:YAG Laser

Incisional biopsy technique was performed using an Er:YAG laser at the following parameters: pulse mode, 35 Hz, 7 W, 200 mJ. Tissue samples were taken along the borders of the lesions, avoiding areas with excess fibrin coating as well as ulcerative fields due to epithelial absence. Biopsies were stored in 10% formalin at neutral pH (6.8–7.2) (biopsy/solution ratio 1:10) until embedded in paraffin. Hematoxylin and eosin (H&E)-stained glass slides were then prepared for histological examination. Laboratory time: 7–10 days.

### 2.3. PBM Therapeutic Protocol

Fourteen days after the first biopsy, all 20 OLP patients started PBM with a diode laser (Syneron 810 nm). The course of treatment was one month, with sessions performed three times per week, every other day (12 treatment sessions in total). The protocol applied was as follows: CW; 0.50 W; 30 s; 1.2 J/cm^2^. The procedure was non-contact with a laser-tissue distance of 2–3 mm. Large lesions were irradiated repeatedly, with overlapping beam spots to cover the entire surface. Two days after the last 12th treatment session, the second biopsy was taken.

### 2.4. Assessment Tools

#### 2.4.1. Clinical Evaluation

Pain and clinical scores of the OLP lesions were evaluated at baseline and the end of treatment (D30).

Pain was assessed by applying a visual analogue scale (VAS) consisting of a line numbered from 0 to 10, where “0” represents “no pain” and “10”—terrible pain, respectively. Each patient was instructed to mark the value that best matched the intensity of pain during the evaluation. The values were then categorized as follows: score 0 = no pain: (VAS = 0); score 1: mild pain (0 < VAS ≤ 3); score 2: moderate pain (3 < VAS ≤ 7); score 3: severe pain (7 < VAS ≤ 10).

The clinical presentation of OLP was evaluated according to the Thongprasom Sign Scoring System (TSSS) [33], and OLP lesions were categorized into scores as follows: score 0 (no lesions), score 1 (hyperkeratotic lesions), score 2 (atrophic area ≤ 1 cm^2^), score 3 (atrophic area > 1 cm^2^), score 4 (erosive area ≤ 1 cm^2^), and score 5 (erosive area > 1 cm^2^). The size of the atrophic and erosive areas was measured with a periodontal probe in “mm”.

#### 2.4.2. Cytokine Evaluation/ELISA Technique/

Cytokine concentrations present in saliva samples were measured using commercial kits (ELISA MAX(TM) Deluxe Human Kits, Biolegend, Germany). IL-1β (Cat. No. 437004, sensitivity—0.5 pg/mL); IL-6 (Cat. No. 430504, sensitivity—4 pg/mL), and TNF-α (Cat. No. 430204, sensitivity—2 pg/mL) were analyzed according to the manufacturer’s protocols. In summary, in 96-well plates pre-treated overnight with the respective capture antibodies, 100 µL of the standards and samples was pipetted per well. After incubation and washing, a biotinylated secondary detection antibody was added, which formed a complex with the primary antibody. After washing, avidin-peroxidase was added to the wells. The addition of the substrate solution—tetramethyl benzoate (TMB)—resulted in a blue coloration. Finally, the reaction was quenched with a stop solution (H_2_SO_4_) whereupon the color of the liquid turned yellow. Absorbance was read spectrophotometrically on an ELISA reader at a wavelength of 450/570 nm. Cytokine concentration was determined based on a standard curve and measured in picograms.

#### 2.4.3. Histological Examination

Hematoxylin–eosin-stained histological sections were made from the biopsies taken before therapy and were used to confirm the diagnosis of OLP and to record the baseline tissue status. Modified WHO criteria of Van der Meij [12] were applied to obtain a histological diagnosis of OLP. At the end of treatment (D30), control biopsies were taken from all OLP patients to analyze the tissue changes that have occurred in response to PBM therapy. Semi-quantitative and semi-qualitative methods were used. Improvement in the morphological picture was considered if at least one of the following criteria was found:

- Reduction in the hyperplastic processes (hyperkeratosis, hypergranulosis and acanthosis) in the epithelium.
- Reduction in the inflammatory infiltrate.
- Recovery of tunica epithelialis in cases with primary atrophic and erosive forms of OLP.
- Recovery of epidermal-dermal attachment in cases with primary bullous form of OLP

### 2.5. Statistical Analysis

Statistical analysis was performed using SPSS 11.5 Inc., Chicago, IL, USA, Excel 7.0 VB for applications, and GraphPad Prism 3.0 (GraphPad Soft, San Diego, CA, USA). After checking the distribution for normality of the variables, the Wilcoxon matched-pairs test was chosen to determine the difference in pain level and the Mann–Whitney test—to determine the difference in lesion sign scores before and after therapy. Differences in the salivary levels of IL-1β, IL-6, and TNF-α between the groups were evaluated by parametric Student’s t-test in case of normal distribution of experimental results. Variation analysis—one-way ANOVA was also used. The results are expressed as an arithmetic mean value ± standard deviation: Mean ± SDM. *p* values less than 0.05 were considered statistically significant.

## 3. Results

### 3.1. Patients Characteristics

A total of 20 patients with OLP met the inclusion criteria and were recruited for this study. Of them, 17 were women and 3 were men, whose ages ranged from 24–73 years old (mean age 52,9). All patients showed more than one OLP lesion, so a total of 95 lesions were recorded. Reticular OLP was the most common clinical presentation (30%), followed by atrophic (25%) and erosive (25%). The other three forms (plaque-like—10%; papular—5%; bullous—5%) were less frequently observed. The lesions were more often multiple and disseminated, with a bilateral and symmetrical distribution.

### 3.2. Clinical Outcomes

Regardless of the clinical form, all participants included in the study felt some degree of pain and discomfort before therapy (score 3—10%; score 2—45%; score 1—45%). Laser irradiation reduced the subjective symptoms in all patients, and five of them (25%) reported a complete disappearance of complaints (score 0). After treatment, there were no patients with severe pain (score 3). The difference in pain level before and after therapy was statistically significant (*р* < 0.0001).

At baseline, a total of 95 OLP lesions were recorded, of which 31.6% were erosions more or less than 1 cm^2^ (scores 5 and 4); 28.4% were atrophic fields more or less than 1 cm^2^ (scores 3 and 2), and 40% were keratotic lesions (score 1). After one month of treatment with an 810 nm diode laser, 25% of the lesions healed completely. At D30, the distribution of the lesions was as follows: 3.2%—score 5; 4.2%—score 4; 8.4%—score 3; 9.5%—score 2; and 49.5%—score 1. The statistical analysis performed showed a significant improvement in the clinical presentation of OLP after PBM therapy (*p* < 0.05).

None of the patients reported side effects.

### 3.3. Salivary Levels of IL-1β, IL-6, TNF-α

**Figure 1:**
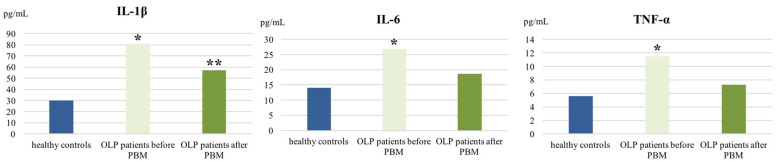
Salivary levels of IL-1β, IL-6, and TNF-α in healthy controls and oral lichen planus (OLP) patients at baseline (D0) and at the end of photobiomodulation (PBM therapy (D30). * Statistically significant difference between healthy controls and OLP patients before therapy and ** between healthy controls and OLP patients after therapy.

According to the study results, salivary levels of IL-1β (80.86 ± 34.3 рg/mL), IL-6 (26.89 ± 10.34 рg/mL), and TNF-α (11.48 ± 6.58 рg/mL) in OLP patients were significantly higher compared to those in healthy controls (30 ± 10.8 pg/mL, 14.06 ± 6.22 pg/mL, and 5.55 ± 3.65 рg/mL, respectively) (Figure 1). Among the cytokines analyzed, IL-1β was observed to be the most concentrated one. After PBM therapy, a reduction in the concentrations of all these pro-inflammatory cytokines was observed (IL-1β—57.26 ± 24.8 pg/mL; IL-6—18.64 ± 6.53 pg/mL and TNF-α—7.31 ± 4.30 рg/mL), although the differences in their levels before and after therapy were not statistically significant. However, salivary concentrations of IL-6 and TNF-α after therapy did not differ significantly from those in healthy subjects either.

### 3.4. Histological Results

At baseline, the following histological findings were recorded: hyperkeratosis—in 80% of the sections; parakeratosis—in 45%; hypergranulosis—in 45%; acanthosis—in 30%; epithelial atrophy—in 15%; liquefaction degeneration in the basal cell layer—35%; Civatte bodies—20%; dense band-like inflammatory infiltrate, consisting mainly of lymphocytes and confined to the superficial lamina propria—in 85%; moderate band-like inflammatory infiltrate at dermoepidermal junction—in 10%; dense band-like polymorphic inflammatory infiltrate—in 5%; epidermolysis (subepithelial detachment)—in 5% of the sections. Еpithelial dysplasia was not detected (0%).

**Figure 2:**
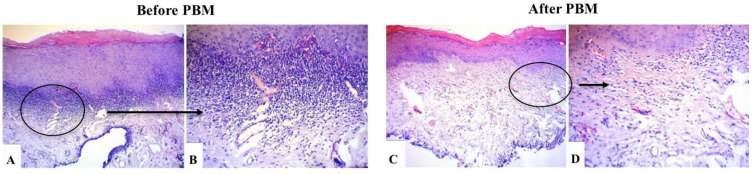
Histological improvement in OLP patient after PBM therapy, H&E staining. Before therapy (**A**): magnification ×10, epithelial hyperplasia (hyperkeratosis, parakeratosis, acanthosis) and dense band-like inflammatory infiltrate in superficial lamina propria and (**B**): magnification ×20, inflammatory infiltrate composed mainly of lymphocytes. After therapy (**C**): magnification ×10, normalization of the thickness of tunica epithelialis with mild hyperkeratosis and parakeratosis; and (**D**) magnification ×20, scattered solitary inflammatory cells and granulation tissue. Histological changes inconsistent with the diagnosis of OLP.

**Figure 3:**
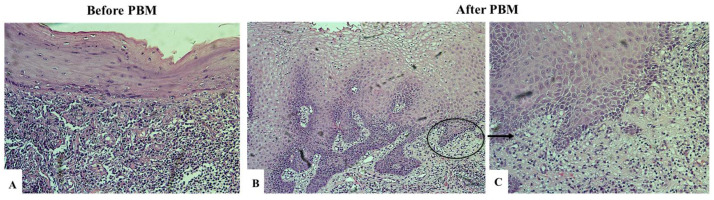
Histological sections of a patient with an atrophic form of OLP before and after PBM therapy, H&E staining. Before PBM therapy (**A**): magnification ×20, stratified squamous epithelium with parakeratosis and epithelial atrophy and dense band-like inflammatory infiltration by lymphocytes and plasma cells. After PBM therapy (**B**): magnification ×10, epithelial hyperplasia and reduction in the inflammatory infiltrate and (**C**): magnification ×20, scattered mild inflammatory infiltrate.

Histopathological changes in lamina epithelialis of OLP lesions after PBM (Table 1):

A predominant finding in the epithelium of OLP lesions was hyperplasia. In most cases, epithelial thickening involved all suprabasal layers and presented as hyperkeratosis, parakeratosis, hypergranulosis, and acanthosis. Deletion of some or all of these pathological processes was observed in 30% of control biopsies taken at the completion of PBM therapy (Figure 2). Conversely, in another 15% (*n* = 3), an increase in the thickness of the epithelial compartment was found (Figure 3). Restored epidermal–dermal attachment in response to laser irradiation was revealed in the only case demonstrating epidermolysis before therapy.

Histopathological changes in lamina propria of OLP lesions after PBM (Table 1):

The inflammatory infiltrate in OLP has a well-defined characteristic—dense, band-like, consisting mainly of lymphocytes and limited to the superficial lamina propria. A reduction, up to complete resolution of the inflammatory infiltrate, was observed in half (50%) of the OLP sections after therapy (Figure 2 and Figure 3).

No cases were registered with data on the occurrence of a dysplastic process.

In total, in 60% of the post-treatment OLP biopsies, an improvement in the morphological picture was observed. Correction of the pathological processes was recorded at different levels—in 10% changes were detected only in the epithelium, in another 10%—only in the inflammatory infiltrate. General improvement without complete deletion of the histological signs of the disease was detected in 10% of cases. In 30% of the control histological sections after PBM, the observed changes were insufficient to confirm the diagnosis of OLP.

## 4. Discussion

OLP is a relatively common disease, especially here in Europe [1]. As the lesions are mostly symptomatic, the associated oral pain and/or burning interfere with food and drink intake, increase patients’ anxiety, impair their psycho-emotional health, and generally worsen the quality of life (QoL) of the patients. In addition, OLP is recognized as a potentially malignant disorder (OPMD) [4]. Therefore, the treatment of this disease is an extremely responsible task. Gold-standard corticosteroid therapy is associated with adverse effects, and moreover, there are lesions refractory to this treatment. PBM has recently been shown to be a promising treatment approach for patients with OLP.

Since OLP is a potentially malignant disease, a concern has arisen as to whether PBM laser irradiation may increase the risk of cancer development. Furthermore, other forms of phototherapy, such as UVA and UVB light, which have been widely applied in the past in the treatment of OLP patients, are of limited use nowadays precisely because of their reported oncogenic potential [2]. Indeed, PBM can enhance tumor cell proliferation [34]. Sperandio et al. demonstrated that LLLT increased cell proliferation in oral dysplastic and oral cancer cell lines and significantly modified the expression of proteins related to progression and invasion (pAkt, pS6, and Cyclin D1) and thus could aggravate oral cancer cellular behavior [35]. Conversely, there are articles in the literature, showing that patients subjected to PBM had better progression-free survival and a tendency for better overall survival [7,36]. However, aside from the above, there is no evidence of malignant transformation of non-dysplastic cells after PBM irradiation. A recent review article addressing the benefits and limitations of PBM therapy in premalignant oral lesions concluded that this treatment modality is a promising way to minimize the clinical symptoms of these lesions. The paper did not provide any data on the potentially harmful effects of using PBM in patients with OLP [37]. In fact, most authors working on the subject define this therapy as safe, with no visible side effects [13]. In a thorough review of the literature, we found no reports examining the rate of malignant transformation of OLP lesions after PBM treatment. This gap may be explained by the inability to obtain multiple follow-up biopsies from the same patient to assess cellular changes and the occurrence of dysplasia. In this regard, saliva currently represents an appealing liquid biopsy for the early diagnosis of oral cancer. Several studies have revealed the potential of using salivary samples for detection of specific circulating miRNAs (miR-31, miR-21, miR-181b, miR-345, etc.) for monitoring the progression of premalignant lesions into malignant ones [38]. This noninvasive diagnostic method offers a new perspective for future research on this issue. However, in the present study, we recorded no adverse effects during the treatment course. In addition, after a one-month treatment with an 810 nm diode laser, we took control biopsies to reveal the resulting cellular changes. No pathological changes or signs of dysplasia were detected. Based on these results, we can conclude that PBM with an 810 nm diode laser is a safe treatment method for OLP patients.

Recently, the effectiveness of PBM therapy in the treatment of OLP has been the focus of extensive research. In most of the studies, the results generally followed the same trend: PBM therapy significantly reduced symptoms and lesion size in patients with OLP [7].

In the present study, 810 nm laser therapy led to subjective symptoms relief in all OLP patients. These results are consistent with those of the aforementioned studies and may be attributed to the ability of PBM irradiation to reduce pain intensity [7,13]. Moreover, our findings demonstrated a significant improvement in the clinical presentation of OLP after PBM therapy. Atrophic-erosive lesions responded better to the applied laser light with a reduction in lesion size or complete resolution, while keratotic forms showed mild or no improvement. This can be explained by the biological activity of PBM irradiation to promote proliferation of keratinocytes and fibroblasts, matrix synthesis, angiogenesis, and vascular remodeling, which are considered key factors in the healing process of the oral mucosa [7].

To claim that a given therapy is effective, we need to understand its specific mechanisms of action. Тherefore, in the present study, we evaluated the effectiveness of PBM in the treatment of OLP also at molecular and histomorphological levels.

The abundance of pro-inflammatory cytokines in OLP lesions contributes to the initiation and perpetuation of the disease. Hence, modulation of cytokine levels is a desirable therapeutic effect. IL-1β is a potent pro-inflammatory cytokine secreted by basal keratinocytes and recruited lymphocytes in OLP lesions. A higher concentration of IL-1β in the saliva of OLP patients has been reported in the literature [27]. Moreover, IL-1β has been shown to be associated with higher clinical OLP scores and erosive lesions, suggesting that it may contribute to disease severity [3]. In this regard, Yamamoto et al. reported that overexpression of IL-1β led to the release of TNF-α, IL-6, and granulocyte-macrophage colony-stimulating factor (GM-CSF), indicating that this cytokine may be responsible for the amplification of the inflammatory cytokine repertoire in OLP [39]. We found significantly increased salivary levels of IL-1β in OLP patients compared to healthy controls (*p* < 0.001). Furthermore, among the cytokines analyzed, IL-1β demonstrated the highest concentration. All this confirmed the role of this cytokine in the pathogenesis of the disease. In a study by Abboud CS et al., IL-1β secretion decreased significantly in response to clobetasol propionate 0.05% treatment, but PBM therapy did not show modulation of these cytokine levels in OLP patients [3]. In contrast, our results demonstrated a marked, although not statistically significant, reduction (80 ± 34 рg/mL—57.29 ± 24.8 рg/mL) in IL-1β in the saliva of OLP patients treated with diode laser.

Several studies have reported higher serum and salivary levels of IL-6 in patients with OLP, especially in those with an erosive/atrophic form of the disease, suggesting that this cytokine may be a marker for monitoring the disease [25,30]. Rhodus et al. showed a significant decrease in IL-6 levels after a 6-week course of 0.1% dexamethasone oral rinse [25]. In contrast, this cytokine was not modulated after treatment with PBM or clobetasol propionate 0.05% in the study by Abboud CS et al. [3]. We found that the concentration of IL-6 in the saliva of OLP patients was significantly higher compared with that in healthy donors (*p* < 0.001) and decreased after PBM therapy. The established reduction was not statistically significant; however, the post-treatment IL-6 levels were very close to those of healthy subjects, with no significant difference between these two groups.

Tumor necrosis factor-α (TNF-α) is one of the prominent cytokines involved in the T-cell immune reaction in OLP, regulating lymphocyte recruitment and cytokine release from other immune cells. Furthermore, the importance of TNF-α in the pathogenesis of the disease is determined by its role in the activation of programmed cell death. Current evidence suggests that CD8+ T-Ly trigger keratinocyte apoptosis in OLP by several mechanisms, one of which is binding of CD8+ T-cell-secreted TNF-α to its specific TNF-α receptor 1 (TNFR-1) on the keratinocyte surface [5]. In addition, it has been previously demonstrated that its high concentration is associated with worst OLP clinical presentation [3]. Rhodus NL. et al. showed that the gold standard corticosteroid therapy was effective in reducing TNF levels in OLP patients [25]. In contrast, PBM therapy with a 970 nm diode laser [25] and a 660 nm diode laser [3] demonstrated little or no modulation of this pro-inflammatory cytokine, respectively. In the present study, TNF-α was found to be significantly increased in the saliva of OLP patients compared to healthy subjects, which is consistent with the results of other studies [28,29]. PBM irradiation for one month resulted in a reduction in its concentration. No significant differences were found between pre- and post-therapy TNF-α levels, nor between post-treatment levels in OLP patients and controls for the same marker. This means that PBM can reduce the concentration of this pro-inflammatory cytokine to a level close to that of health.

The simultaneous reduction in IL-1β, IL-6, and TNF-α levels at the end of the 810 nm diode laser treatment course confirmed the effectiveness of PBM therapy in reducing inflammation in OLP patients.

Histologically, OLP demonstrates typical but not pathognomonic features. The difficulty in making a reliable histological diagnosis in OLP stems from the following factors: there is great diversity of histopathological findings, depending on the clinical form; no unified diagnostic criteria for the disease have been adopted; and many of the typical microscopic features of OLP, such as Civatte bodies and so-called interface mucositis (dense band-like inflammatory infiltrate consisting mainly of lymphocytes and confined to the superficial lamina propria), can also be seen in other diseases [10]. There is a group of disorders, such as contact OLR, drug-induced OLR, and OLR associated with Graft-versus-host disease (GVHD); chronic ulcerative stomatitis (CUS), lichen planus pemphigoides, proliferative verrucous leukoplakia, etc., that histopathologically resemble OLP [10].

In the present study, pre- and post-therapy biopsies were taken for histological analysis from all 20 OLP patients using the Er:YAG laser. Some advantages of the latter could be mentioned, including simplified procedure, better visibility of the operative field due to the use of air/water spray removing the blood, significantly shorter duration of the intervention (*p* < 0.001) [40], and significantly better incision regularity (*p* < 0.001) [41] compared to the use of scalpel. Regarding the thermal effect of the Er:YAG laser, almost all studies addressing the topic concluded that the observed areas of tissue damage do not hinder the histological diagnosis [40,41,42]. Moreover, in all the studies that compared the Er:YAG laser with other laser systems, the Er:YAG laser showed the least thermal effect [42]. All this determines the Er:YAG laser as an instrument of choice for excisional and incisional biopsies of oral lesions, being an adequate alternative to the cold scalpel [40,41,42]. In the present study, no specimens demonstrating extensive areas of thermal tissue damage limiting histological analysis were recorded.

As discussed earlier in this article, there are three sets of diagnostic criteria for OLP: the WHO criteria, Van der Meij modified WHO criteria, and those proposed by the American Academy of Oral and Maxillofacial Pathology [10]. In the present study, we applied the WHO criteria, modified by Van der Meij [12]. In the latter, the authors introduced the term “histopathologically compatible with OLP” for all cases that do not meet all the listed criteria. However, in reality, as shown in a study by Sanches et al., only a small proportion of histological sections diagnosed as OLP demonstrated all diagnostic criteria [43]. For example, the authors reported the absence of basal layer degeneration, Civatte bodies, and even inflammatory infiltrate in 60.4%, 31.2%, and 25.0% of the samples they analyzed, respectively (*n* = 48) [43]. In this regard, only 35% of the cases in the present study fulfilled all diagnostic criteria. Liquefaction degeneration was absent in 65% of sections, and in another 15%, the inflammatory infiltrate did not show the exact characteristic for OLP—the density of the infiltrate was moderate to low (10%) or the inflammatory infiltrate was polymorphic (5%). In addition, no significant association between lesion type (clinical form) and histological and morphometric variables was reported by Sanches et al. [43]. In line with this, although five of the patients included in our study were clinically diagnosed with an erosive form of OLP, erosions were not detected in histological sections, and only three of five patients with an atrophic form showed thinning of tunica epithelialis on microscopic examination of their tissue biopsies. This discrepancy may be attributed to the biopsy technique for removing keratotic tissues (pathognomonic Wickham’s striae), avoiding areas of ulceration, as the latter often lack an epithelial compartment. Since most of the typical features of OLP, such as Civatte bodies and keratinocyte liquefaction degeneration, are found in the basal epithelial cell layer, the latter must be present to ensure a histological diagnosis.

Given that the diagnosis of OLP is based on clinico-pathological concordance, the assessment of treatment outcomes should provide both clinical and histological data. For a therapy to be defined as effective in patients with OLP, it must also correct the typical histological finding of the disease. There is a paucity of studies that investigate histological improvement in response to treatment. After extensive research into the literature, we found only a few articles that correspond to this topic. Gambino et al., 2021, compared the biostimulatory effect of 980 nm diode laser to 0.05% clobetasol propionate in patients with symptomatic atrophic-erosive OLP. As an assessment tool, they used optical coherence tomography (OCT), which provides a real-time, non-invasive, tissue investigation. After 8 weeks of treatment, the authors reported a significant increase in the width of the stratified epithelium (*p* < 0.05) and a significant decrease in the width of lamina propria (*p* < 0.05) in both groups [44]. These results may be explained as a consequence of the ability of PBM and clobetasol to promote epithelial healing and reduce interface inflammation [44]. The second related article, entitled “Histopathological features of oral lichen planus and its response to corticosteroid therapy. A retrospective study” [45] evaluated the histopathological features of OLP, but only at the time of diagnosis, and correlated them with the clinical course of the disease and the response to corticosteroid therapy. The authors reported that from all the analyzed histopathological variables of OLP, only the presence of plasma cells in the band-like infiltrate of T lymphocytes was significantly associated with lower OLP exacerbations and a better response to corticosteroids [45]. However, they did not compare pathomorphological findings before and after therapy, which is what we intended.

To the best of the authors’ knowledge, this is the first study to evaluate histological changes after PBM therapy in patients with OLP. We found an improvement in the histological features of the disease in 60% of the sections, but at different levels. In the epithelial compartment, the laser irradiation most commonly (30%) resulted in a partial reduction in the hyperplastic processes. A single case of squamous stratified epithelium without pathological processes was achieved. Still, clinically, most of the keratotic lesions demonstrated mild or no improvement. Conversely, epithelial thickening was found in three of the control specimens after therapy (15%). However, all of them were initially diagnosed with an atrophic form of the disease. The thinned epithelium is deficient in terms of its barrier function and predisposes to pain sensitivity. Laser irradiation appears to stimulate regenerative mechanisms, which leads to an increase in the thickness of the epithelial covering. This may explain the relief of subjective symptoms reported by the three patients. In the only case that presented at the time of diagnosis with subepithelial epidermolysis, a restored epidermal–dermal attachment was found after PBM therapy. We consider this effect to be particularly beneficial since the bullous form of lichen planus is the most severe manifestation of the disease, and reestablishment of dermo-epidermal adhesion is a basic requirement for improving the condition of these patients. Of course, reliable conclusions cannot be made based on a single unit of observation, but these results suggest that this treatment modality could be effectively applied to patients with severe bullous dermatoses such as pemphigus and pemphigoid.

In 50% of the control biopsies after therapy, a reduction in the inflammatory infiltrate was detected. This finding, along with the established decrease in the analyzed pro-inflammatory cytokines, determines the ability of PBM to manage the inflammatory process in OLP.

In 30% (*n* = 6) of the tissue sections after therapy, the observed changes were insufficient to confirm the diagnosis of OLP. In fact, there was only one patient who demonstrated histological healing and complete clinical improvement—absence of pain and disappearance of the oral lesions. In three cases, this seeming “cure” was characterized by the disappearance of the inflammatory infiltrate and the growth of granulation tissue, and in another two—a scanty residual infiltrate was present, which, however, did not show the distinctive characteristics of OLP. Although the diagnosis of OLP cannot be made under these circumstances, mild epithelial hyperplasia was found in these specimens. All these five patients were clinically diagnosed with keratotic forms of OLP and showed moderate (*n* = 2), mild (*n* = 2), and no improvement (*n* = 1) after one month of laser therapy. Keratinocytes often respond to the presence of underlying inflammation by thickening of the epithelial layers. The reported results showed that laser irradiation leads to the elimination of the lymphocytic infiltrate in the lamina propria but does not significantly affect the hyperplastic processes in the epithelium. Therefore, it is likely that the laser light is able to affect the inflammatory response but is inefficient in removing its consequences—once keratotic epithelium has occurred, it remains so. In this regard, it can be speculated that in the absence of inflammation, subsequent mechanical exfoliation of the pathologically thickened epithelium by other means, such as laser ablation, would lead to the achievement of a clinically healthy mucosa. Applied to one of the patients, this treatment protocol demonstrated an excellent response.

## 5. Conclusions

In the present study, we applied PBM therapy with an 810 nm diode laser to patients with different forms of symptomatic OLP. The obtained results defined this treatment method as safe, not associated with side effects or pathological cellular changes. The analgesic and wound-healing effects of PBM were confirmed, as a significant reduction in symptoms and clinical signs of the disease was achieved. In addition, the simultaneous decrease in IL-1β, IL-6, and TNF-α levels at the end of the treatment course proved the ability of the laser therapy to reduce inflammation in OLP patients. To the best of the authors knowledge, this is the first study to report histological improvement after treatment of OLP lesions, characterized by partial correction of pathological processes in the tunica epithelialis and reduction to complete resolution of the inflammatory infiltrate. Thus, in the present study, the effectiveness of PBM therapy in OLP patients was confirmed at the clinical, molecular, and histomorphological levels. Further randomized controlled clinical trials with an increased number of patients are needed to confirm the validity of the results obtained.

## Figures and Tables

**Table 1:** Histopathological changes in OLP lesions after PBM.

	Tissue Change	OLP Tissue Sections	
		*n*	%
Lamina epithelialis			
	Epithelial hyperplasia reduction	6	30%
	Epithelial thickening	3	15%
	Epidermal-dermal attachment repair	1	5%
	No improvement	10	50%
Lamina propria			
	Inflammatory infiltrate reduction	10	50%
	No improvement	10	50%

## Data Availability

The original contributions presented in the study are included in the article, further inquiries can be directed to the corresponding authors.
